# Necrotizing Soft Tissue Infections Caused by Morganella morganii: A Case Report and Review of the Literature

**DOI:** 10.7759/cureus.80718

**Published:** 2025-03-17

**Authors:** Masakazu Kakurai, Shigehiko Takeyama, Yoshihiro Moriyama

**Affiliations:** 1 Dermatology, Tsuchiura Kyodo General Hospital, Tsuchiura, JPN

**Keywords:** morganella morganii, necrotizing fasciitis, necrotizing soft tissue infections, skin and soft tissue infections, surgical debridement

## Abstract

*Morganella morganii* is an unusual opportunistic pathogen often isolated as a cause of nosocomial infections in adults, particularly in wound and urinary tract infections. Herein, we present a case of necrotizing soft tissue infections in the right lower leg to the foot caused by *Morganella morganii*. A 74-year-old Japanese male with a history of microscopic polyangiitis and chronic kidney disease was referred to our department with severe painful swelling, warmth, and purpura extending from the right lower leg to the foot. An exploratory incision revealed a discharge of a large amount of light yellowish pus, and the superficial fascia and overlying fat tissue were easily dissected using a swab. Surgical debridement was performed. Pus and two sets of blood cultures grew only *Morganella morganii*. Taken together, necrotizing soft tissue infections caused by *Morganella morganii* were diagnosed. Based on the results of the bacterial antibiotic susceptibility testing, antibiotic therapy was changed from intravenous meropenem to ciprofloxacin hydrochloride hydrate. Although skin necrosis did not spread after surgical debridement, the patient’s general condition gradually deteriorated, and the patient died 14 days after hospitalization. This case is notable as the patient developed necrotizing soft tissue infections caused by *Morganella morganii*. We also reviewed eight reported cases of necrotizing soft tissue infections caused by *Morganella morganii*, including our case.

## Introduction

*Morganella morganii* (*M. morganii*) is a facultative anaerobic, rod-shaped, Gram-negative enteric bacterium often associated with nosocomial infections in adults, specifically postoperative wound and urinary tract infections [1,2]. Its virulence and increasing drug resistance contribute to high mortality rates in some *M. morganii* infections [1,2]. Diseases caused by *M. morganii* include urinary tract infections, bacteremia, sepsis, septic arthritis, wound infections, cellulitis, and necrotizing soft tissue infections (NSTIs) [1,2]. The frequency of skin and soft tissue infections is relatively high in *M. morganii* infections [1,2]. However, NSTIs caused by *M. morganii* are rare, and no comprehensive literature exists to date.

NSTIs are rapidly progressing, life-threatening bacterial infections of soft tissue, resulting in widespread tissue destruction that may extend from the epidermis to the deep musculature [3]. This infection can occur after major traumatic injuries, minor breaches of the skin, or non-penetrating soft tissue injuries. The annual incidence of NSTIs ranges from 0.3 cases to 15.5 cases per 100,000 population [3]. NSTIs are largely distinguished by whether the cause is polymicrobial (type Ⅰ) or monomicrobial (type Ⅱ) [3]. Type Ⅰ infections are polymicrobial infections involving aerobic and anaerobic pathogens, whereas type Ⅱ infections, a monomicrobial infection, are mainly caused by group A *Streptococcus* [3]. Other less common type Ⅱ pathogens include *Aeromonas hydrophila*, *Vibrio vulnificus*, *Bacteroides*, and *Escherichia coli* [3].

We herein present a case of NSTIs extending from the right lower leg to the foot caused by *M. morganii* and review eight patients with *M. morganii* NSTIs, including ours.

## Case presentation

**Figure 1:**
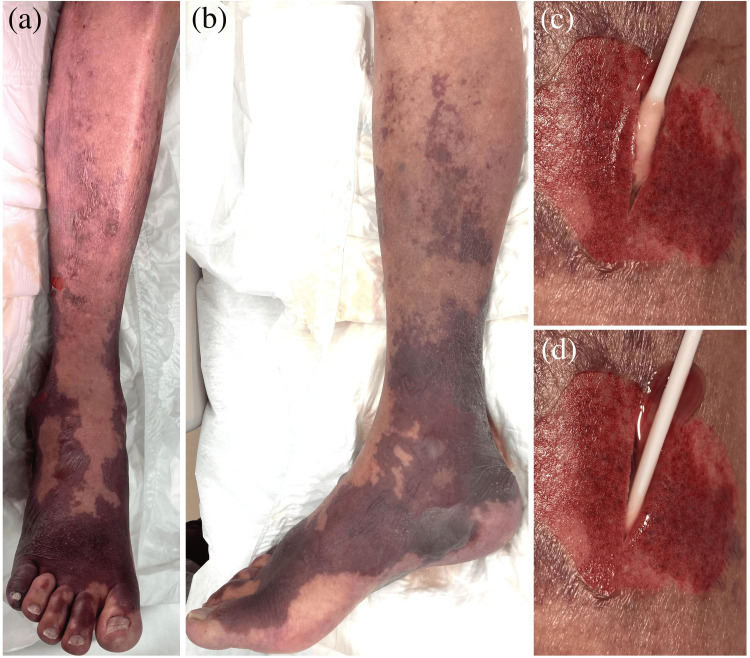
Cutaneous findings. (a, b) Severe pain, swelling, and warmth with irregularly shaped purpura accompanied by bullae extending from the right lower leg to the foot. (c, d) Discharge of a large amount of light yellowish pus during exploratory incision. The superficial fascia and overlying fat tissue were easily dissected using a swab.

A 74-year-old Japanese male presented to the emergency department with a two-day history of right lower leg pain that limited his ability to perform activities of daily living. On admission, he had a fever, chills, and right lower leg pain without warmth, swelling, or erythema. Intravenous cefepime dihydrochloride hydrate was initiated with a provisional diagnosis of cellulitis; however, during the following 36 hours, purpura developed from the right lower leg to the foot, and he was referred to our department. His medical history included microscopic polyangiitis and chronic kidney disease (CKD) on hemodialysis, and his medications included prednisolone (5 mg/day), vonoprazan fumarate, and sulfamethoxazole-trimethoprim. On examination, the patient had an axillary body temperature of 39.6℃, a heart rate of 90 beats per minute, a blood pressure of 92/61 mmHg, an oxygen saturation of 96% on room air, and a respiratory rate of 20 breaths per minute. The patient appeared unwell but showed no signs of impaired consciousness. Physical examination revealed severe, painful swelling and warmth, with irregularly shaped purpura accompanied by bullae extending from the right lower leg to the foot (Figures 1A, 1B). Skin lesions were limited to this region, with no skin ulcers; however, a skin fissure measuring 1 cm was observed on the right heel. Peristaltic sounds were normal, and no abdominal or costovertebral angle tenderness was noted. Blood tests revealed a white blood cell count of 3.22 × 10^3^/μL, hemoglobin of 10.5 g/dL, a platelet count of 6.3 × 10^4^/μL, blood urea nitrogen of 31.4 mg/dL, creatinine of 5.33 mg/dL, creatine kinase of 4934 U/L, C-reactive protein level of 30.50 mg/dL, prothrombin time-international normalized ratio of 1.68, and fibrinogen degradation products of 57.0 μg/mL (Table 1). Contrast-enhanced computed tomographic images ruled out acute limb ischemia and deep vein thrombosis. An exploratory incision was made to the depth above the superficial fascia on the right lower leg, which resulted in a discharge of a large amount of light yellowish pus with a slight odor; the superficial fascia and overlying fat tissue were easily dissected using a swab (Figures 1C, 1D).

**Table 1:** Laboratory data of blood samples. PT-INR: prothrombin time-international normalized ratio; FDP: fibrinogen degradation products.

Blood test	Reference value (male)	On admission	On day 2	On day 10
Aspartate aminotransferase (U/L)	8-38	21	168	113
Alanine aminotransferase (U/L)	4-44	10	35	21
Lactate dehydrogenase (U/L)	124-222	471	660	1148
Sodium (mEq/L)	135-147	142	140	139
Potassium (mEq/L)	3.6-5.0	4.9	4.3	4.1
Urea nitrogen (mg/dL)	8-20	43.9	31.4	47.7
Creatinine (mg/dL)	0.61-1.04	7.46	5.33	5.19
Creatine kinase (U/L)	57-197	109	4934	725
C-reactive protein (mg/dL)	0-0.20	2.03	30.50	26.22
White blood cell (/μL)	4,000-9,000	3,050	3,220	12,930
Hemoglobin (g/dL)	14.0-18.0	9.6	10.5	10.5
Platelet (×10^4^/μL)	15.0-35.0	7.6	6.3	3.1
Hemoglobin A1c (%)	4.6-6.2	5.0	–	–
PT-INR	0.90-1.10	0.97	1.68	1.13
FDP (μg/mL)	0-5.0	–	57.0	47.5
D-dimer (μg/mL)	0-1.0	6.2	17.3	18.9

**Figure 2:**
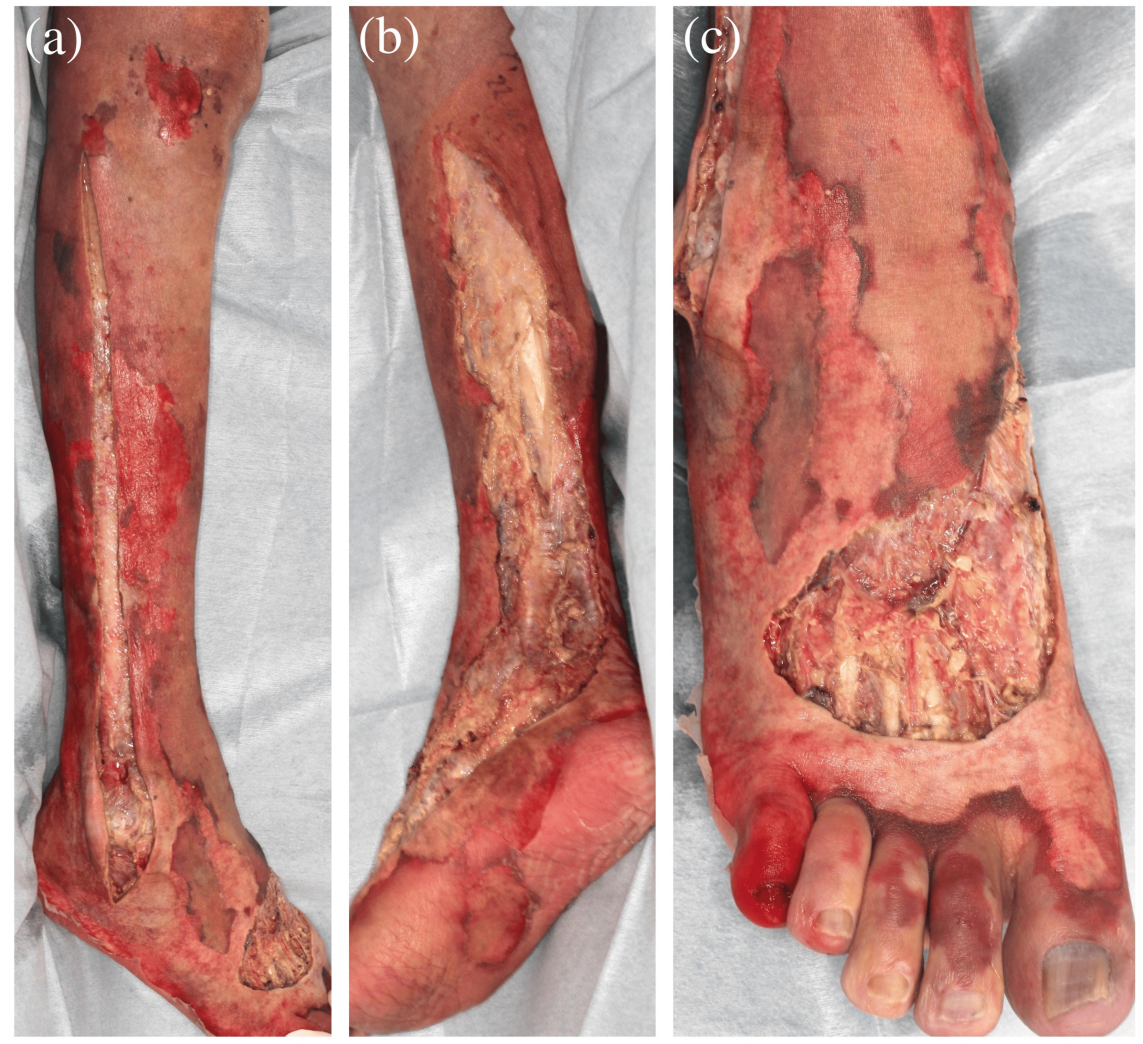
Postoperative cutaneous images. The necrotic skin was removed from the right lower leg to the foot.

Because the patient refused amputation, surgical debridement was performed from the right lower leg to the foot (Figure 2). The wounds were left open for drainage.

Postoperatively, the patient received circulatory and respiratory support in the intensive care unit, and cefepime dihydrochloride hydrate was changed to meropenem. On day three of hospitalization, two sets of blood cultures taken on admission grew *M. morganii* alone, whereas Gram staining of the urine did not reveal any microorganisms, and the urine culture was negative. Additionally, pus cultures grew only *M. morganii*. Therefore, the diagnosis of NSTIs caused by *M. morganii* was made. On day six, meropenem was switched to intravenous ciprofloxacin hydrochloride hydrate based on antibiotic susceptibility testing (Table 2).

**Table 2:** Antimicrobial susceptibility of the Morganella morganii obtained from blood and pus cultures. MIC: minimum inhibitory concentration; S: susceptible; R: resistant.

Antimicrobial agent	MIC (μg/mL)	MIC interpretation
Ampicillin	>16	R
Cefazolin	>16	R
Ceftriaxone	≤1	S
Cefepime	≤2	S
Ampicillin-sulbactam	>16	R
Piperacillin-tazobactam	≤8	S
Meropenem	≤0.12	S
Levofloxacin	≤0.12	S
Gentamicin	>8	R
Sulfamethoxazole-trimethoprim	>2	R

Although skin necrosis did not spread after surgical debridement, the patient’s general condition gradually worsened, and the patient died 14 days after hospitalization.

## Discussion

*M. morganii* is a facultative anaerobic, rod-shaped, Gram-negative enteric bacterium found in the environment and intestinal tracts of humans, mammals, and reptiles as part of the normal flora [1,2]. Initially considered a cause of summer diarrhea and an insignificant pathogen, it is now recognized as an important pathogen, mainly responsible for nosocomial infections in adults, particularly postoperative wound and urinary tract infections [1,2]. A typical example of animal-to-human transmission is *M. morganii*-associated skin and soft tissue infections following snakebites [4]. Although case reports of *M. morganii* infections are scattered, infections are most commonly reported in Taiwan, Japan, the United States, and Spain [1].

Diseases caused by *M. morganii* are diverse and include urinary tract infections, pneumonia, peritonitis, pericarditis, meningitis, bacteremia, sepsis, septic arthritis, osteomyelitis, pyomyositis, wound infections, cellulitis, and NSTIs [1,2]. In a previous study of 136 patients with *M. morganii* infections, skin and soft tissue infections were the most common (19%), followed by sepsis (11%), abscess (10%), urinary tract infections (8%), and bacteremia (7%) [1]. In another study on patients with *M. morganii* bacteremia, skin and soft tissue infections were the most common sources of bacteremia, followed by primary bacteremia and urinary tract infections [2]. Therefore, the frequency of skin and soft tissue infections is relatively high among *M. morganii* infections, and the skin is one of the portals of entry. However, NSTIs caused by *M. morganii* are rare and poorly reported, and no comprehensive literature exists.

NSTIs are a life-threatening bacterial infection of the soft tissue and are largely distinguished by whether the cause is polymicrobial (type Ⅰ) or monomicrobial (type Ⅱ) [3]. Type Ⅰ infections are polymicrobial infections involving aerobic and anaerobic pathogens that usually occur in the elderly or those with underlying medical conditions, including diabetes mellitus (DM). Type Ⅱ infections are mainly caused by group A *Streptococcus* and often occur in healthy individuals of all ages [3]. Other less common type Ⅱ pathogens include *Aeromonas hydrophila* and *Vibrio vulnificus* [3]. In addition, monomicrobial NSTIs caused by Gram-negative pathogens such as *Bacteroides* and *Escherichia coli* have been reported. This may not align with the typical patient characteristics of type Ⅱ infections, as it mainly occurs in immunocompromised patients [3]. Similarly, in our case, an immunocompromised patient was infected by *M. morganii* alone and developed NSTIs.

To date, only eight patients with NSTIs caused by *M. morganii*, including ours, have been reported (Table 3) [5-10].

**Table 3:** Reports of necrotizing soft tissue infections caused by Morganella morganii. F: female; M: male; NA: not available; DM: diabetes mellitus; HT: hypertension; MPA: microscopic polyangiitis; CKD: chronic kidney disease.

Author/year	Age/sex	Medical history	Affected sites	Other detected bacteria	Surgical treatment	Course
Krebs et al. (2001) [5]	0/F	Low birth weight, prematurity	Left leg	Escherichia coli	Above knee amputation	Dead
Lee et al. (2009) [6]	3/M	NA	Right ankle	*Enterococcus* species	Debridement	Improve
Soleimanian et al. (2011) [8]	67/M	DM	Right leg and right trunk	Escherichia coli, Proteus mirabilis, Enterococcus faecalis, Citrobacter freundii, Providencia rettgeri	Debridement	Improve
Richards et al. (2015) [7]	81/M	HT, heart failure, atrial fibrillation, stroke	Right lower extremity	Aeromonas hydrophila	Above knee amputation	Improve
Leiblein et al. (2020) [9]	64/M	Gastric bypass, cholecystectomy, appendectomy	Left groin	Proteus mirabilis, Enterococcus faecalis, Bacteroides fragilis	NA	Improve
Soedjana et al. (2024) [10]	NA	NA	NA	Klebsiella pneumoniae	Debridement	Dead
	NA	NA	NA	None	No surgery	Dead
Our case	74/M	MPA, CKD	Right lower leg and foot	None	Debridement	Dead

Among the six patients with documented age, onset ranged from 0 to 81 years, occurring in children up to three years old and the sixth and eighth decades of life. Five of the six patients were male. All five patients with a documented medical history had prematurity, DM, CKD, or multimorbidity. Our case was the only one involving immunosuppressant use (prednisolone). The affected sites were located on the lower extremities (6/6 patients) and trunk (1/6 patients), excluding the upper extremities and head and neck region. In three patients, including ours, the cause of NSTIs was presumed to be skin entry [5,6]. Another patient developed an NSTI after a peripheral bypass of the lower extremity [7]. In the remaining four patients, the entry of *M. morganii* was unknown [8-10]. Six out of eight patients had polymicrobial infections [5-10], and the remaining two patients, including ours, had monomicrobial infections [10]. In summary, NSTIs caused by *M. morganii* tend to affect the lower extremities of pediatric and elderly males who are immunosuppressed or have multiple morbidities. Most cases of *M. morganii* NSTIs are polymicrobial infections, but monomicrobial NSTIs of *M. morganii* can occur.

Although early aggressive surgical intervention is important in the initial treatment of NSTIs to reduce mortality [3,10], the prognosis varies widely depending on factors such as the pathogen and host conditions. In a study of 90 patients with NSTIs in which most of the causative pathogens (85%) were Gram-negative bacteria, the overall mortality rate was 13%, and the highest mortality rate was in the nonsurgical group (37%, 3/8 patients), followed by surgical debridement alone (29%, 8/28 patients) and debridement followed by skin grafting (4%, 1/24 patients) [10]. In our literature review, half of the patients with *M. morganii* NSTIs died despite surgical debridement or amputation and appropriate antibiotic therapy, suggesting a poor prognosis.

*M. morganii* is normally susceptible to third- and fourth-generation cephalosporins, carbapenems, and quinolones, whereas it has intrinsic resistance to most first- and second-generation cephalosporins and ampicillin [1,2], consistent with our antibiotic susceptibility testing results. Recently, drug resistance has increased in *M. morganii*, resulting in high mortality rates in some *M. morganii* infections [1,2]. Additionally, *M. morganii* has been identified as a significant cause of nosocomial infections [1,2]. Therefore, appropriate antibiotic therapy and nosocomial infection control measures (e.g., hand washing and personal protective equipment) are important when *M. morganii* infections occur.

The limitations of this study include the limited number of cases and missing data from several reports. Additionally, among the polymicrobial NSTIs in which *M. morganii* was detected, its degree of contribution to infection remains unclear. Further accumulation of *M. morganii* NSTI cases is required to fully understand the pathogenesis, epidemiology, clinical characteristics, and appropriate treatments to reduce mortality.

## Conclusions

Our case is unique as the patient developed NSTIs due to *M. morganii*, an unusual pathogen. To date, eight patients with *M. morganii* NSTIs, including ours, have been reported. Our review indicates that *M. morganii *NSTIs primarily affect the lower extremities of pediatric and elderly males with immunosuppression or multimorbidity. Most cases of *M. morganii* NSTIs are polymicrobial infections, but monomicrobial NSTIs of *M. morganii *can occur, as in our case. Half of the patients died despite surgical debridement or amputation, suggesting a poor prognosis. Further case accumulation is necessary to advance our understanding of its pathogenesis, epidemiology, and clinical characteristics, as well as to optimize treatment strategies.

## References

[1] Liu H, Zhu J, Hu Q, Rao X (2016). Morganella morganii, a non-negligent opportunistic pathogen. Int J Infect Dis.

[2] Erlanger D, Assous MV, Wiener-Well Y, Yinnon AM, Ben-Chetrit E (2019). Clinical manifestations, risk factors and prognosis of patients with Morganella morganii sepsis. J Microbiol Immunol Infect.

[3] Stevens DL, Bryant AE (2017). Necrotizing soft-tissue infections. N Engl J Med.

[4] Tsai YH, Hsu WH, Huang KC, Yu PA, Chen CL, Kuo LT (2017). Necrotizing fasciitis following venomous snakebites in a tertiary hospital of southwest Taiwan. Int J Infect Dis.

[5] Krebs VL, Koga KM, Diniz EM, Ceccon ME, Vaz FA (2001). Necrotizing fasciitis in a newborn infant: a case report. Rev Hosp Clin Fac Med Sao Paulo.

[6] Lee CY, Lee HF, Huang FL, Chen PY (2009). Haemorrhagic bullae associated with a chicken scratch. Ann Trop Paediatr.

[7] Richards CR, Clark ME, Bowen DK, Uratake D, Ayubi F, Katras T, Kellicut DC (2015). Necrotizing soft tissue infection following a peripheral bypass. Ann Vasc Surg.

[8] Soleimanian S, Gordon NC, Wareham DW (2011). Polymicrobial necrotizing fasciitis involving enterobacteria producing CTX-M-15 extended-spectrum β-lactamases. J Med Microbiol.

[9] Leiblein M, Wagner N, Adam EH, Frank J, Marzi I, Nau C (2020). Clostridial gas gangrene - a rare but deadly infection: case series and comparison to other necrotizing soft tissue infections. Orthop Surg.

[10] Soedjana H, Christine S, Sisca F (2024). Treating necrotizing fasciitis patients at the topmost referral hospital in West Java, Indonesia: 6 years experience. Int Wound J.

